# Properties of Sweet Buttermilk Released from the Churning of Cream Separated from Sheep or Cow Milk or Sheep Cheese Whey: Effect of Heat Treatment and Storage of Cream

**DOI:** 10.3390/foods11030465

**Published:** 2022-02-05

**Authors:** Lambros Sakkas, Vasiliki Evageliou, Panagiotis E. Igoumenidis, Golfo Moatsou

**Affiliations:** 1 Department Food Science and Human Nutrition, Agricultural University of Athens, 75 Iera Odos, 11855 Athens, Greece; lasakkas@hotmail.com (L.S.); evageliou@aua.gr (V.E.); 2 Department of Food Science and Technology, University of West Attica, 28 Agiou Spiridonos, 12243 Egaleo, Greece; igoumenidisp@gmail.com

**Keywords:** sheep/cow buttermilk, whey buttermilk, frozen cream, thermized/pasteurized cream, antioxidant properties, minerals, phospholipids, functional properties

## Abstract

The objective of the study was to compare the buttermilk released from the churning of sweet cream separated from sheep milk (BSM) or whey (BSW) with the buttermilk from sweet cow milk cream (BCM). Additional experimental factors were the heat treatment (68 °C for 10 or 30 min) and storage of cream (refrigeration or freezing). The composition of BSM was the most advantageous in terms of non-fat solids, protein—which was the most abundant solid component—casein, calcium and phosphorus contents. No significant differences were observed in the phospholipids (PL) content of BSM, BCM and BSW. Antioxidant potential and emulsion stability (ES) of BSM were the highest. The radical scavenging activity (RSA) of BSW was high opposite to chelating activity (CA). Some functional properties of BSW were similar to those of BSM and BCM. The freezing of cream affected the churning, the fat content, the soluble nitrogenous fraction at pH 4.6 (WSN) and some functional properties of buttermilk, but not in a consistent manner. The properties of BSM were marginally affected or unaffected by the use of frozen cream. The freezing of whey cream caused significant changes (*p* < 0.05) in the protein profile and the functional behaviour of BSW. Cream heat treatment affected the WSN of BSW opposite to its sweet cream counterparts.

## 1. Introduction

Sweet or acidified buttermilk is the by-product of the butter making process and contains most of the ingredients of milk fat globule membrane (MFGM) and relatively small amounts of triglycerides, depending on the butter making conditions [1]. The non-acidic pH of sweet buttermilk is appropriate for most food formulae and is the main type of commercial buttermilk [2,3,4]. The composition of sweet buttermilk is similar to skim milk in terms of gross composition and its major components are the non-fat components of churned cream, i.e., caseins, serum/whey proteins, lactose and minerals [2,5]. The dry matter ranges from 8 to 12% and the fat on dry matter from 4.6 to 14.5% [3]. Buttermilk contains parts and components of MFGM, such as proteins and polar lipids [3,6,7,8,9,10], in quantities affected by the conditions of the heat treatment and churning of cream [2,5,11,12]. The concentration of polar lipids can be 2% on dry matter or 80–125 mg per g fat of buttermilk, that is much higher than the respective 0.28% of skim milk and 2.7–4.8 mg per g of full-fat milk [6,12]. Due to the phospholipids (PL), buttermilk exhibits emulsifying properties, considerable water holding capacity and lower foaming capacity than skimmed milk [2,13,14]. The biological properties of buttermilk and MFGM, which are mainly attributed to sphingomyelin, PL, sialic acid and gangliosides, are remarkable. Protective activity against infections and bacterial toxins, reduction in cholesterol levels, anticancer and antioxidant potential, regulatory role at the cellular level, positive effect on the development and function of nerve tissue, hair and skin, prevention of age-related cognitive decline and muscle loss, have been reported [4,6,12,15,16,17,18,19,20].

The multi-functionality of buttermilk is exploited in several applications of food technology, e.g., for the improvement of water holding capacity and texture and the protection against lipid oxidation of bakery, confectionary and sauce-type products [3,4]. In the dairy industry, buttermilk is used for the production of beverages, as an ingredient of ice cream, as an additive to increase the thermal stability of reconstituted condensed milk and for the improvement of structure, sensory characteristics and yield of low-fat yogurt and cheese [3,11,21,22,23,24,25]. Components of buttermilk and MFGM are involved in the formation of liposomes for the delivery of drug or food additives. Finally, due to the presence of sphingomyelin, which is not present in plant polar lipids, buttermilk ingredients are used in infant formulae and cosmetics [8,19,20].

There is a limited number of publications on the properties and technological behaviour of buttermilk or MFGM preparations from differently treated cream or from different types of cream of cow origin. It has been reported that the increase in the intensity of cream heat treatment decreases the soluble proteins [5,24] and increases the fat content [26,27] or the percentage of PL in fat of buttermilk [5]. It affects adversely the rennet clotting behaviour of milk supplemented with buttermilk or the rheological properties of the resultant gel [5,24,26,27]; although, a similar effect was not observed in milk supplemented with MFGM fractions with variable thermal history [28]. Sweet cow cream and whey cream buttermilk differ in respect to protein profile, lactose content and organoleptic characteristics [29], protein content [30] or colour [31]. Whey buttermilk exhibits greater emulsifying and lower foaming ability compared with its sweet cream counterpart [32], while lower emulsifying capacity in MFGM fractions from whey-derived buttermilk has been observed [32].

Publications for buttermilk from non-cow milk are scarce, despite the technologically interesting features of their lipid fraction and structural elements [33,34,35]. In brief, the mean diameter of sheep milk fat globule (MFG) ranges within 3–3.8 μm, which is lower than the mean diameter of cow MFG. The concentration of total polar lipids expressed as percentage of milk fat ranges within 0.38–0.70 and 0.36–0.82%, for sheep and cow MFG, respectively. There are also differences in the structural characteristics of the MFGM. There is a higher contribution of monounsaturated and a lower contribution of saturated fatty acids in the polar lipid fraction of sheep milk (SM) compared with cow milk (CM). Average cholesterol content of sheep and cow MFGM is 1.8 and 1.7 mg/m^2^, respectively. Sheep MFGM contains more sphingomyelin than cow MFGM and has a lower cholesterol to sphingomyelin ratio, i.e., 2.8 and 3.6 mol/mol, respectively. Gangliosides in the outer surface of sheep MFGM are considerably less than in CM. The diameter of casein micelle of SM is greater than that of CM, i.e., 170–220 nm and 150–180 nm, respectively, and the most abundant casein in SM is β-casein [33,34,35].

Hamad et al. [36] investigated the use of goat and sheep buttermilk in the manufacture of the fermented dairy product Jameed. Lamothe et al. [37] found that the PL to protein ratio was higher in goat buttermilk than in its cow counterpart. Parrón et al. [38] reported similar protein profile and lactose and protein contents for cow and sheep buttermilk, but higher lipid content for the latter. The same group [38,39,40] reported a higher dose-dependent antiviral activity of sheep buttermilk and its components compared with its cow counterpart. The gross composition and cheese making potential of sweet sheep and goat buttermilk alone or in mixtures with milk with variable fat content has been investigated by Sakkas et al. [24]. The same group [25] studied the properties and ripening course of semi-hard cheese from reduced-fat SM supplemented with sweet sheep buttermilk.

The aim of the present study was to examine the effect of cream origin and treatment on the compositional, physical and functional properties of sweet buttermilk. The main subject was the sweet buttermilk released during the churning of cream separated from SM or sheep cheese whey (SW). The experimental factors were the heat treatment of cream—thermization or pasteurization—and the storage of heat-treated sweet cream before low-temperature ripening and churning, that is, immediate use or freezing for five months. For comparison, sweet buttermilk from CM was manufactured and analysed under the same conditions. Among the new elements of the present work are the study of SW buttermilk, the use of frozen cream and the comparative presentation of the effect of manufacturing conditions on buttermilk resulting from different kinds of cream.

## 2. Materials and Methods

### 2.1. Experimental Design and Sampling Scheme

**Figure 1:**
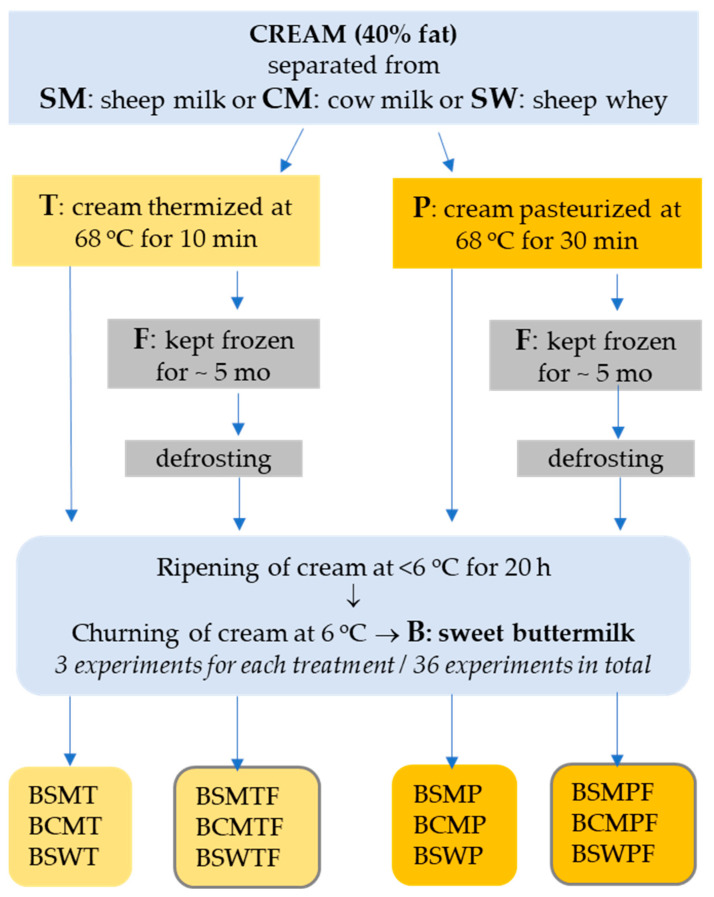
Design of the experiments, types of manufactured buttermilks and codes of collected buttermilk samples. BSMT—buttermilk from SM-thermized cream; BCMT—buttermilk from CM-thermized cream; BSWT—buttermilk from SW-thermized cream; BSMP—buttermilk from SM-pasteurized cream; BCMP—buttermilk from CM-pasteurized cream; BSWP—buttermilk from SW-pasteurized cream. F at the end of a buttermilk code indicates the use of frozen cream.

The design of the experiments, the types of manufactured buttermilks and the codes of collected samples are schematically presented in Figure 1. In detail, the experiments were performed as follows:

- Preparation of creams: Creams were obtained by skimming thermized SM and CM and by double skimming pasteurized SW. SW was collected from the manufacture of a hard-type cheese made from curd heated up to 50 °C. All cream types were standardized to 40% fat and were divided in two equal portions of 2.5 kg each. The first portion was thermized at 68 °C for 10 min and the second was pasteurized at 68 °C for 30 min. After rapid cooling down to 10 °C, creams were either ripened at <6 °C for 20 h or kept frozen at <−20 °C for five months (frozen creams). Three replicates were performed for each combination of the experimental factors (CM, SM and SW cream, thermized or pasteurized cream, fresh or frozen cream).
- Buttermilk from non-frozen cream: After ripening at low temperature, creams were churned to butter and buttermilk at 6 °C using an overhead mixer. Buttermilk was filtrated with sterilized cloth-filter before collection and coded as follows: BCMT—buttermilk from CM-thermized cream; BCMP—buttermilk from CM-pasteurized cream; BSMT—buttermilk from SM-thermized cream; BSMP—buttermilk from SM-pasteurized cream; BSWT—buttermilk from SW-thermized cream; BSWP—buttermilk from SW-pasteurized cream.
- Buttermilk from frozen cream: Frozen creams were defrosted at 6 °C within 2 days and remained at room temperature for 2 hours before heating in a water bath at 30 °C under gentle stirring for a few minutes. Then, remained at room temperature and stirred periodically, till they reached 24 °C (CM, SM) or 16 °C (SW). Finally, all types of cream were rapidly cooled down to 10 °C and ripened at <6 °C for 20 h prior to churning. The temperature sequence for frozen creams had been developed in preliminary experiments. Churning of creams to butter and buttermilk took place at 6 °C using an overhead mixer. Buttermilk was collected after filtration and coded as follows: BCMTF—buttermilk from CM-thermized and frozen cream; BCMPF—buttermilk from CM-pasteurized and frozen cream; BSMTF—buttermilk from SM-thermized and frozen cream; BSMPF—buttermilk from SM-pasteurized and frozen cream; BSWTF—buttermilk from SW-thermized and frozen cream; BSWPF—buttermilk from SW-pasteurized and frozen cream.

### 2.2. Analysis of Milk, Whey, Cream and Buttermilk

The gross composition of milk, whey and buttermilk was estimated by the FT-IR analyser Milkoscan-FT120 (Foss, Hillerød, Denmark). Titratable acidity and pH of milk, whey, cream and buttermilk were determined by means of the Dornic method and a pH meter, respectively [41]. Fat content of milk, whey and buttermilk was estimated by the Gerber method [42]. The fat content of cream was determined in a 1:1 mixture of cream and distilled water using a “Koehler” butyrometer.

### 2.3. Analysis of Buttermilk Nitrogenous Fractions

Protein and acid-soluble protein of buttermilk were determined in duplicate by the Kjeldahl method. Buttermilk water (acid)-soluble nitrogen fraction (WSN) was prepared by acidification at pH 4.6 and total nitrogen (TN) and WSN contents were assessed in duplicate by the Kjeldahl method [41]. The concentration of major whey proteins in buttermilks was estimated by analysis of WSN in duplicate by means of reversed-phase high-performance liquid chromatography (RP-HPLC) as described by Sakkas et al. [24].

### 2.4. Biofunctional Potential of Buttermilk

#### 2.4.1. Minerals

The determination of calcium, sodium, potassium and magnesium contents in the ash fraction of buttermilks was performed by the atomic absorption spectrometric method (AAS) [43]. The phosphorus content of buttermilk was assessed by molecular absorption spectrometry (MAS) [44]. The stock dilution for AAS analysis was 25 mg of buttermilk ash in one mL of 25% (*v*/*v*) HNO_3_ made up to 100 mL with Milli-Q water. The stock dilution for the MAS analysis was 25 mg of buttermilk ash in 2.5 mL of HCl (36 g/L) made up to 100 mL with Milli-Q water.

#### 2.4.2. Antioxidant Activity

Two different assays were used for the evaluation of buttermilk antioxidant activity. 2,2-diphenyl-1-picrylhydrazyl (DPPH∙) radical scavenging activity (RSA) was determined in triplicate [45], in a mixture of 150 mg of buttermilk or methanol (control) or Trolox and 850 μL of 0.1 mM DPPH∙ in methanol. The determination of Fe^2+^-chelating activity (CA) was performed in triplicate as described by Moschopoulou et al. [45], adjusting the concentration of the buttermilk in the mixture to that reported by Conway et al. [46]. Buttermilk was diluted 1:75 in Milli-Q water, centrifuged (1000× *g* for 10 min) and the supernatant was filtered through 0.45 μm poly(vinylidene fluoride) Whatman filter (PVDF). Then, 700 μL of filtrate or ultra-pure water (control) or ethylenediaminetetraacetic acid (EDTA) were mixed with 70 μL of 1.2 mM FeSO_4_ in Eppendorf tubes. After thorough stirring, the mixture was incubated for 30 min in the dark, at room temperature, and 70 μL of 2.4 mM ferrozine was added. Tubes were vortexed for 2 min and 240 μL were transferred in 96-well microplates. After stirring for 3 min, an absorbance at 562 nm was recorded.

#### 2.4.3. Phospholipids (PL)

The buttermilk fat extraction was performed as described by Sakkas et al. [25] with some modifications: 15 g of buttermilk, 15 mL of hexane and 10 mL of isopropanol were used, the final mixture was centrifuged at 1000× *g* for 10 min at 20 °C, re-extraction of the bottom layer was not performed and the duration of holding at 102 °C was 30 min. The determination of PL content was performed according to Sakkas et al. [25].

### 2.5. Particle Size Distribution in Buttermilk

Buttermilks were heated at 40 °C for 60 min prior to analysis with a SALD-2300 Shimadzu laser diffraction particle size analyser (Shimadzu Corporation, Tokyo, Japan). Buttermilks were dispersed in a sampler water bath where a laser beam was transmitted. The intensity and the angle of the scattered light emitted by the particle groups were detected and used for the calculation of particle size distribution.

### 2.6. Functional Properties of Buttermilk

#### 2.6.1. Sample Preparation

Prior to measurements, all samples were brought to 3% *w*/*w* protein concentration with either dilution with distilled water (sweet cream buttermilks) or evaporation (whey cream buttermilks). Frozen whey buttermilk samples (−4 °C) were freeze dried under constant vacuum conditions at −10^5^ Pa and −40 °C for 24 h using a UNICRYO MC2L (Munich, Germany) apparatus. For viscosity measurements, evaporation of whey buttermilk samples was performed by means of an IKA Labortechnik Rotary Evaporator RV 06-ML (Staufen, Germany) at temperatures below 45 °C. Measurements for all studied functional properties were performed in two pH values, namely 4.5 and 6.6. pH was adjusted by 0.1 and 1 M HCl or NaOH solutions.

#### 2.6.2. Viscosity

Viscosity of all samples at both pH values was measured at room temperature (20 °C) with an Anton Paar MCR 102 Rheometer (Anton Paar GmbH, Graz, Austria), using a cone–plate geometry. The cone had a diameter of 50 mm and an angle of 1°, while the gap between the cone and the plate was set at 0.101 mm. The temperature of each buttermilk sample, on the rheometer plate, was equilibrated at 20 °C for 5 min prior to measurement. Shear rates from 1 to 100 s^−1^ were applied during each measurement, to obtain a flow curve. For clarity reasons, the apparent viscosity values presented (in mPa·s) correspond to 100 s^−1^ rate. Three measurements were performed for each sample and mean viscosity values were calculated.

#### 2.6.3. Emulsifying Properties

Oil-in-water (o/w) emulsions were prepared by mixing 10 mL of corn oil and 40 mL of each sample at room temperature using a CAT X 120 homogeniser (M. Zipperer GmbH, Ballrechten-Dottingen, Germany) at 22,000 rpm for 2 min. Then, 10 mL of each emulsion were transferred to a 15 mL centrifuge tube. The tube was sealed tightly and stored at 4 °C. The determination of the emulsion’s stability (ES) was based on its height change due to phase separation during storage, as described by Huang et al. [47]. Thus, the initial height of each emulsion upon preparation (H_0_) and the height of the remaining emulsified layer volume after one day of storage (H_storage_) were recorded. ES was determined by the following equation: ES (%) = (H_storage_/H_0_) × 100. For each pH, three emulsions were formed and measured.

#### 2.6.4. Foaming Properties

For foam formation, 30 mL (V_s_) of each sample at both pH values were stirred with a CAT X 120 homogeniser (M. Zipperer GmbH, Ballrechten-Dottingen, Germany) at 22,000 rpm for 3 min and then transferred into a graduated cylinder. The initial volume of the foam (V_f_) was measured, and foaming ability (FA) was calculated as follows: FA (%) = V_f_/V_s_. Measurements were repeated three times per pH value.

#### 2.6.5. Protein Solubility

Each sample at both pHs was centrifuged at 12,000× *g* for 15 min at 25 °C (Z 326 K, Hermle Labortechnik GmbH, Wehingen, Germany). The supernatant was collected and analysed for its protein content with the Kjeldahl method. Protein solubility (PS) was calculated by the following equation: PS (%) = (protein content of supernatant/3) × 100.

### 2.7. Statistical Analysis

Thirty-six buttermilks were analysed and statistical analysis of the data was carried out using the Statgraphics Centurion XVI (Manugistics, Inc., Rockville, MA, USA). The effects of cream origin, cream heat treatment and cream storage and their interactions were assessed by multifactor analysis of variance (ANOVA). The significant differences between means were investigated by the least significance method (LSD) and a significance value of 0.05. Correlation coefficients amongst variables were investigated by means of simple linear regression.

## 3. Results and Discussion

### 3.1. Manufacturing Parameters

The average composition of raw SM used for the preparation of cream was as follows: fat 6.21 ± 0.59%, protein 5.43 ± 0.17%, lactose 4.64 ± 0.05%, acidity 19.08 ± 1.01% lactic acid (LA) and pH 6.68 ± 0.04. The respective parameters for raw CM were as follows: fat 3.34 ± 0.30%, protein 3.11 ± 0.05%, lactose 4.43 ± 0.07%, acidity 13.54 ± 0.97% LA and pH 6.66 ± 0.06. The composition of SW was as follows: fat 1.37 ± 0.17%, protein 1.12 ± 0.03%, total solids 8.96 ± 0.27%, acidity 9.65 ± 0.60% LA and pH 6.44 ± 0.04.

The conditions of heat treatment did not affect the acidity and the pH of the cream. Τhe pH of sheep, cow and whey cream was 6.76 ± 0.04, 6.79 ± 0.09 and 6.46 ± 0.05, respectively. The acidity expressed as % LA was 0.091 ± 0.002, 0.074 ± 0.003 and 0.068 ± 0.007, respectively. The pH was not in accordance with % acidity, due to the interference of caseins in the estimation of the latter. Both the acidity and the pH of whey cream were statistically significantly lower (*p* < 0.05) than those of cream prepared from milk.

The average initial temperature of all cream types, i.e., 36 experiments, was 5.8 ± 0.20 °C. Table 1 is a synopsis of the manufacturing conditions/parameters for the various types of buttermilks of the present study grouped according to cream origin and preservation. The heat treatment of cream—thermization or pasteurization—had no significant effect. The cream origin and storage and their interaction affected statistically significantly (*p* < 0.05) the churning duration (min), the final temperature at the end of churning and the buttermilk yield, expressed as percentage of the quantity of the churned cream. The duration of churning is an important parameter of butter making and affects the composition of buttermilk [1]. The churning time (min) of CM cream was significantly higher (*p* < 0.05) compared with both types of cream of sheep origin. Churning duration was strongly positively correlated with the cream’s pH (*r* = 0.900) but the opposite was true for the % acidity (*r* = 0.383). The use of frozen cream decreased dramatically (*p* < 0.05) the churning duration and in turn decreased the yield statistically significantly (*p* < 0.05). Churning starts with the disruption of the MFGM. Apparently, the temperature sequence applied in frozen cream had destabilized the surface of MFG prior to churning without any visible change in cream structure.

### 3.2. Compositional Parameters

Compositional parameters of various types of buttermilk grouped according to cream origin and preservation are shown in Table 2; the heat treatment of cream was not a statistically significant factor (*p* > 0.05).

Buttermilk composition was statistically significantly affected (*p* < 0.05) by the cream origin. The interaction of cream origin and cream preservation affected significantly (*p* < 0.05) the majority of compositional parameters especially those of CM buttermilk. The significant (*p* < 0.05) decrease in pH in SW buttermilk and in buttermilks from frozen creams can be assigned to manufacturing particularities such as the lower initial pH and the double skimming of whey cream or the additional temperature sequence applied in frozen cream.

Since all cream types had similar fat content and initial temperature, an increase in fat content in buttermilk coincides with high liquid fat content in cream during churning, rapid churning or small-sized MFG [1]. The higher fat content of SM buttermilks from non-frozen SM and SW cream was in accordance with the smaller size of sheep MFG [34,35] and the faster breaking down compared with its cow counterpart (Table 1). The fat content of buttermilks was substantially changed by the use of frozen cream but not in the same direction for the three types of buttermilk. The fat content of CM buttermilk increased opposite to those of sheep origin. The rapid churning of frozen cream (Table 1) is expected to increase the fat content [1], as observed in the BCMF in the present study (Table 2). On the contrary, under the same conditions, the fat content of BSMF and BSWF, that is, of buttermilks of sheep origin, decreased. Τhe different behaviour of both types of sheep frozen creams indicates the involvement of factors that are specific for SM fat. Interestingly, SM lacks agglutinins that induce flocculation of MFG [48]. To our knowledge, there is no relevant scientific information for the churning of frozen cream of sheep origin. The significant change (*p* < 0.05) of fat content of buttermilks from frozen creams affected accordingly the concentration of the remaining solids. However, the non-fat solids (SNF) were significantly affected only in CM buttermilk (*p* < 0.05).

As reported earlier, the types of buttermilks of the present study differed significantly in terms of composition although they came from creams with the same fat content. The milk origin also induced statistically significant differences between sweet cream buttermilks (Table 2). The greatest part of total solids of BCM was lactose, with 43.8 ± 5.08% on average—that is, 48.3 ± 1.58 and 39.2 ± 2.13% of total solids, for buttermilk from non-frozen and frozen cream, respectively. Proteins were the second most abundant group of constituents, i.e., 31.7 ± 0.94 and 24.9 ± 1.63% of total solids, respectively. On average, protein and lactose expressed on the SNF of BCM were 34.6 ± 0.69 and 53.6 ± 0.59%, respectively. The composition of sweet buttermilk of cow origin has been estimated in several studies. In general, buttermilk contains 8–12% total solids and 4.6–14.5% lipids on dry matter [3]. Sodini et al. [2] reported that 48.7–53.8%, 31.5–33.5% and 5.7–13.1% of the buttermilk total solids are lactose, protein and lipids, respectively. According to Morin et al. [49], 30.3% and 8.41% of dry matter are proteins and fat, while the same group [30] found that 25% of dry matter consisted of proteins and 12.2% of lipids with a total solid content of 11.74 ± 0.59%. In the study of Gassi et al. [5], the total solids of sweet cream buttermilk ranged from 8.88 to 9.16%, fat from 0.45 to 0.54% and protein from 2.78 to 2.94%. Buttermilk prepared under laboratory conditions in the study of Barry et al. [50] consisted of 11.1% total solids, 2.8% fat, 3.36% protein and 4.23% lactose. Lambert et al. [51] found that the average total solids content of industrial whole buttermilks was low, i.e., 8.7 ± 0.8%, protein 2.9 ± 0.3% and total lipids 0.9 ± 0.4%.

As shown in Table 2, BSM had significantly higher (*p* < 0.05) mean total solids—close to 14%—due to substantially higher protein and fat content compared with its cow counterpart (BCM). In contrast to BCM, proteins were the most abundant group of solid components of BSM. In particular, 40.5% ± 1.33%, 37.7 ± 2.19% and 17.6 ± 3.33% of dry matter were protein, lactose and fat, respectively. Interestingly, 49.2 ± 0.98 and 45.8 ± 1.01% of SNF of BSM were proteins and lactose, respectively. Information for the composition of sheep buttermilk is scarce. Recently, Sakkas et al. [24] studied the cheesemaking properties of sweet cream buttermilk of sheep or goat origin. The average total solids content of SM buttermilk was 17.1% due to a higher fat content (6.85%) compared with the present study; however, SNF were 10.25%. Protein content was 4.96% and lactose 4.80% or 48.4% and 46.8% of SNF similarly to the estimations of the present study. Higher lipid content for sheep sweet buttermilk powder compared with that of cow origin—20% vs. 15.5%—has also been reported by Parrón et al. [38]. In contrast to our findings, they found that protein was lower than lactose content, i.e., 30.5 and 39.5% (*w*/*w*), respectively, and similar to the respective contents of 27.5 and 39% (*w*/*w*) estimated in cow sweet buttermilk powder. Higher concentrations of total solids and proteins have also been reported for sheep buttermilk from acidified cream compared with a similar preparation from CM [36]. The gross composition of the former was 7.8% dry matter, 0.7% fat and 5.10% proteins and the respective contents of the latter were 6.7, 0.25 and 3.25%, respectively.

BSW had the lowest total solids, protein and SNF contents among the three buttermilk types of the present study (Table 2). Mean fat content on dry matter (total solids) of BSW was 17.3 ± 6.1% and similar to 17.6 ± 3.32% estimated for BSM. However, the major solid component of BSW was lactose, which was twice the protein content. Two thirds of SNF was lactose, i.e., protein content was 29.9 ± 0.62% and lactose was 64.5 ± 0.68% of SNF. To our knowledge, there are no reports for buttermilk from SW cream. There are studies that exhibit the differences between the compositional profile of sweet cream and whey buttermilk of cow origin. The dry matter of whey buttermilk has been estimated as 7.61 ± 0.92%, that is two thirds of that of regular sweet buttermilk. Protein was 0.99 ± 0.02%, approximately one third of that of regular buttermilk [30]. In the study of Sodini et al. [2], 63.4% of the dry matter of whey buttermilk was lactose, 15.5% lipids and 15.5% proteins; the latter was half the protein content of the sweet cream buttermilk analysed in parallel. Moreover, the whey buttermilk had a pH of 5.98—lower than pH 6.46–6.61 of the regular buttermilk. Total solids content of whey buttermilk in the study of Costa et al. [52] was 8.05%, on average, and proteins and lipids were 2% and 1.31%, respectively. Approximately 40%, 35% and 11% of the dry matter of sweet cream buttermilk was lactose, protein and lipids, respectively, and the respective contents in whey buttermilk were 60, 20 and 11% [29].

### 3.3. Nitrogenous Fractions

**Figure 2:**
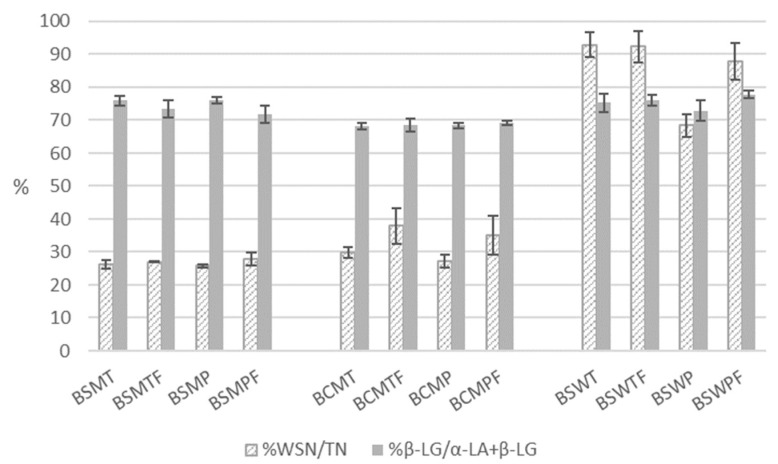
Soluble nitrogenous fraction of various types of buttermilk. WSN—nitrogen soluble at pH 4.6; TN—total nitrogen; β-LG—β-lactoglobulin; α-LA—α-lactalbumin; BSMT—buttermilk from SM-thermized cream; BCMT—buttermilk from CM-thermized cream; BSWT—buttermilk from SW-thermized cream; BSMP—buttermilk from SM-pasteurized cream; BCMP—buttermilk from CM-pasteurized cream; BSWP—buttermilk from SW-pasteurized cream. T—cream thermized at 68 °C for 10 min; P—cream pasteurized at 68 °C for 30 min. F at the end of a buttermilk code indicates the use of frozen cream.

According to multifactor ANOVA, the various expressions of the concentration of nitrogenous fractions of buttermilks were statistically significantly (*p* < 0.05) affected by the three experimental factors of the study (Table 3). Figure 2 is a detailed presentation of the indices of the nitrogenous soluble fraction of the buttermilks.

The churning of frozen cream significantly increased (*p* < 0.05) the nitrogen content of the fraction soluble at pH 4.6 symbolized earlier as WSN and consequently the ratio WSN/TN. Possible explanations could be proteolysis due to additional treatments of cream before churning and changes in the colloidal phase induced by freezing. According to multifactor ANOVA, the pasteurization of cream did not affect the total protein and protein on dry matter contents of buttermilk but significantly decreased (*p* < 0.05) the concentration of WSN. The latter can result from the heat induced denaturation of whey proteins with the exception of α-lactalbumin (α-LA), which tolerates the pasteurization conditions applied in the present study [41]. In fact, the statistically significant effect of heat treatment was due to BSW. Heating conditions did not significantly affect the soluble fraction of sweet sheep or cow cream buttermilks. The mean WSN/TN ratios (*n* = 6) of BSMT and BSMP were 26.6 ± 0.95 and 26.9 ± 1.75%, respectively. The respective percentages for BCMT and BCMP were 33.9 ± 5.7 and 31 ± 5.81%. A statistically significant difference (*p* < 0.05) was observed in the WSN/TN ratios of whey-originated BSWT and BSWP, which were 92.6 ± 3.88 and 78.1 ± 11.355, respectively. The reduction in soluble nitrogen observed in BSWP can be partially assigned to the—not statistically significant—reduction in native β-lactoglobulin (β-LG), which was 8% lower compared with its thermized counterpart. Under heating conditions similar to the pasteurization conditions of the present study (68 °C for 30 min)—which do not coincide with the denaturation of β-LG—β-LG is associated with MFGM proteins via disulphide bonds [53,54]. Most of the original MFGM proteins were involved in interactions with either serum proteins or other MFGM proteins during heating at 65 °C for 30 min [53]. Under the same conditions, approximately 0.3 mg of β-LG per g fat are associated with MFGM, 3 times that estimated for unheated milk [54].

Variable quantities of soluble nitrogen in sweet buttermilk of cow origin have been estimated in various studies. The variability is mainly due to different heat treatments of cream. O’ Connel and Fox [55] estimated the ratio pH 4.6 soluble protein to total protein as 25 ± 3.2% and 24.4 ± 1 for skimmed buttermilk from raw cream and skimmed raw milk, respectively. Ratios of soluble nitrogen to TN of various sweet cream and whey buttermilk powders lower than the findings of the present study, have been presented by Sodini et al. [2]. They report 14.5–21.6% and 60% pH 4.6-soluble nitrogen on TN for cream and whey buttermilk, respectively. The respective ratios for non-protein nitrogen were 6.3–7.6 and 27.1%. In the publication of Gassi et al. [5], the pH 4.6-soluble nitrogen on TN of sweet buttermilk from cream heated at 88–94 °C for 80 s ranged from 36.5 to 32.6% and the non-protein nitrogen was 26.1–28.3% of TN. The concentration of β-LG was from 1600 to 290 mg/kg and that of α-LA from 650 to 330 mg/kg, depending on cream heat treatment—much lower than our findings.

As expected, the insoluble protein fraction at pH 4.6 (INSP) was very low in BSW (Table 3) since cream originated from cheese whey normally contains traces of caseins that have not been retained in the cheese curd. However, INSP in buttermilk does not consist entirely of caseins. Some of the MFGM proteins, such as butyrophilin and xanthine oxidase, along with denatured whey proteins precipitate under acidic conditions [2,55,56]. Caseinomacropeptide (CMP), which is one of the products of rennet-induced clotting, contributed substantially to the soluble fraction of this type of buttermilk in the present study (BSW, Table 3).

### 3.4. Biofunctional Potential

#### 3.4.1. Minerals

The heat treatment of cream did not affect the concentration of major minerals of buttermilks. The effect of cream preservation was statistically significant (*p* < 0.05) only for the phosphorus content of BSW (Table 4). On the contrary, there was a strong influence of the cream origin on the concentration of major minerals in buttermilks.

It is well known that the mineral content and profile of milk is correlated with the protein content and is related to milk origin. Sodium, potassium and chloride are diffusible and two thirds of calcium, one third of magnesium and about half the phosphate are associated with casein micelle. Therefore, it can be expected that the decrease in INSP could decrease major minerals, such as calcium and phosphorus. Potassium and sodium contents that are not associated with casein are in accordance with literature for milk, e.g., up to 140 and 150 mg potassium per 100 mL milk are reported for SM and CM, respectively [35]. Since they are diffusible, they are transferred to the whey, as confirmed in Table 4. The opposite holds true for calcium and phosphorus that are retained in the cheese curd along with paracasein. The phosphorus concentrations in SM and CM are 124–158 and 92–99 mg per 100 mL, respectively. The lower contribution of INSP to the BCM compared with milk can explain the reduced phosphorus and calcium content of the former, as in Table 4.

Calcium content of buttermilks was low (Table 4) considering that SM and CM contain 195–200 and 104–128 mg calcium per 100 mL [35]. Moreover, the calcium to phosphorus ratios in BSM and BCM, both released from the churning of sweet cream, were 0.94 and 0.70, i.e., much lower than the average 1.30 observed in milk. Therefore, the churning selectively reduced calcium.

Low concentration of calcium in buttermilk has been previously presented. Ramachandra Rao et al. [57] estimated 20–22 mM calcium (approx. 80–88 mg/100 mL) in buttermilk that is lower than the 26–32 mM calcium in milk [1]. O’Connell and Fox [55] found lower calcium content in skimmed buttermilk compared with skimmed milk, i.e., 94.8 ± 5.4 and 119.5 ± 9.3 mg per 100 g, respectively. They suggested as a possible explanation the formation of insoluble salts due to complexation of calcium with free fatty acids released into the aqueous phase during the breaking of the oil-in-water emulsion. On the other hand, Gassi et al. [5] reported higher calcium concentration in buttermilks, ranging from 97 to 115 mg/100 g. Nevertheless, the calcium and phosphorus contents were correlated with the INSP, in which casein is included, i.e., r = 0.97 and r = 0.99, respectively.

#### 3.4.2. Antioxidant Activity

The antioxidant potential of various types of buttermilks, by means of two assays, is presented in Table 5. The heat treatment of cream did not affect the results.

The %DPPH∙ RSA was significantly (*p* < 0.05) higher in BSM and BSW compared with BCM. The %DPPH∙ RSA was positively and significantly correlated with the contents of soluble proteins (0.52, *p* = 0.0011) and native whey proteins in buttermilk (CMP, 0.72, *p*= 0.009; α-LA, 0.62, *p* = 0.0001; β-LG, 0.64, *p* = 0.0001). The concentration of whey proteins was significantly higher (*p* < 0.05) in BSM and BSW than in BCM (Table 3). Considering the above and that there was no significant correlation (*p* > 0.05) between %DPPH∙ RSA and INSP, we suggest that DPPH∙ RSA should be attributed mainly to whey proteins and other pH 4.6-soluble nitrogenous compounds. Previously, Conway et al. [46] have associated radical absorbance capacity with peptides derived from whey proteins, mainly α-LA and β-LG; meanwhile, according to Cichosz et al. [58], all β-LG-derived peptides possess RSA. Khan et al. [59] have attributed RSA of whey proteins to amino acids that contain sulphur compounds, whereas Cichosz et al. [58] have marked the significance of peptide secondary structure. Moreover, RSA has been also associated with MFGM proteins by Conway et al. [46], who suggested butyrophilin as the main source of peptides with potent antioxidant effect. Furthermore, the PL fraction of MFGM is considered responsible for antioxidant activity, as they contain poly-unsaturated fatty acids capable of binding cations and ether lipids, which act very effectively against hydroxyl radicals [58].

Both the cream origin and preservation affected significantly (*p* < 0.05) the %Fe^2+^CA. The highest %Fe^2+^ CA was detected in BSM and the lowest in BSW, which exhibited 5-fold lower values compared with the former. Since cheese whey mainly contains whey proteins and no or traces of caseins, the Fe^2+^ CA could be assigned mainly to casein and the peptides derived from it. In the present study, %Fe^2+^ CA correlated positively and strongly (0.95, *p* = 0.000) with %INSP—that is, mostly caseins—and negatively and significantly with %WSN (−0.60, *p* = 0.0001). Conway et al. [46] have attributed Fe^2+^ CA to casein-derived peptides and especially phosphopeptides, which can interact with metals due to the polar side chains of some amino acid parts. This complies with the strong positive correlation (*p* = 0.0000) of %Fe^2+^ CA with % phosphorus (0.92) and % calcium (0.88) in our study, while % INSP was also strongly positively correlated (*p* = 0.0000) with both calcium (0.97) and phosphorus (0.99) contents. A considerable CA for casein, casein fragments and phosphopeptides has been also reported [58,59]. Moreover, %Fe^2+^ CA was higher in BSM compared with its cow counterpart apparently due to its higher casein content.

#### 3.4.3. Phospholipids (PL)

**Figure 3:**
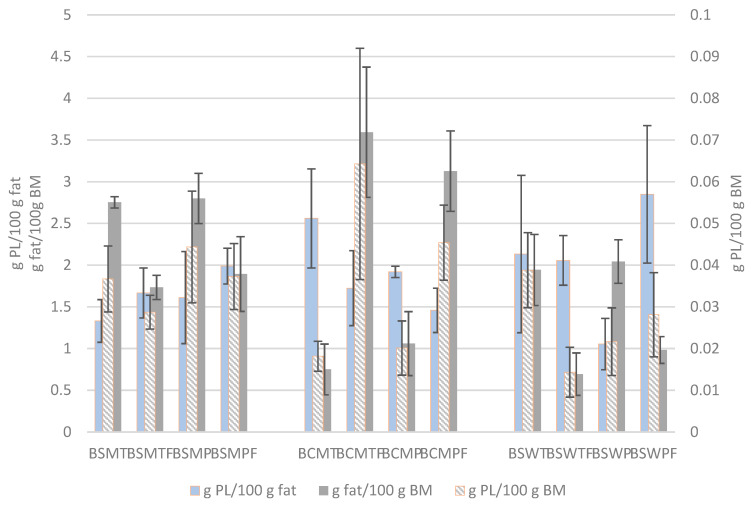
Phospholipid (PL) concentration and fat content of various types of buttermilk. Mean and standard deviation of three experiments. BSMT—buttermilk from SM-thermized cream; BCMT—buttermilk from CM-thermized cream; BSWT—buttermilk from SW-thermized cream; BSMP—buttermilk from SM-pasteurized cream; BCMP—buttermilk from CM-pasteurized cream; BSWP—buttermilk from SW-pasteurized cream. T—cream thermized at 68 °C for 10 min; P—cream pasteurized at 68 °C for 30 min. F at the end of a buttermilk code indicates the use of frozen cream.

The PL content of the buttermilks expressed in g per 100 g buttermilk and in g per 100 g fat are presented in Figure 3. For comparison reasons, the fat contents of buttermilks are also shown. Both expressions of PL concentration were not affected by the experimental factors. The average PL content of BSM, BCM and BSW was 0.037 ± 0.01, 0.037 ± 0.024 and 0.026 ± 0.012 g per 100 g buttermilk, and 1.65 ± 0.39, 1.92 ± 0.54 and 2.02 ± 0.87 g per 100 g fat, respectively. The respective contents expressed on buttermilk total solids were 0.269 ± 0.066, 0.313 ± 0.171 and 0.28 ± 0.13 g per 100 g total solids.

Variable PL concentrations in buttermilk influenced by the manufacturing conditions and expressed in different ways are presented in the literature. Several publications report higher total PL concentrations in untreated buttermilk than our findings, such as 2.1 g per 100 g buttermilk dry matter [49], 0.14 and 0.10 g per 100 g regular and whey buttermilk [30], 0.16 g per 100 g buttermilk or 2.03 g per 100 g buttermilk dry matter [60], 1.27–1.34 or 1.87 g per 100 g regular and whey buttermilk dry matter [2], 0.082–0.112 g per 100 g buttermilk from differently heat-treated creams [5], and 0.99 g per 100 g buttermilk [50]. Similarly to our results, Verardo et al. [61] estimated a concentration of 0.024–0.044 g per 100 g buttermilk, depending on butter making conditions, and Konrad et al. [62] reported 0.03–0.053 g per 100 g whey buttermilk depending on the fat content of whey cream.

Barry et al. [50] showed that different extraction methods result in statistically significantly different PL contents in milk. Morin et al. [26] expressed concern about the interference of complexes among milk proteins, MFGM fractions and PL in the extraction of PL. The association between casein and MFGM in buttermilk is a well-known phenomenon that limits the yield of MFGM-enriched fractions from buttermilk. The addition of sodium citrate before the microfiltration of milk [63], casein precipitation by acid and rennet coagulation [64], or enzymatic hydrolysis of buttermilk proteins before ultrafiltration and diafiltration [62,65]—that can be followed by supercritical extraction using carbon dioxide alone or with ethanol [52,66,67]—have been applied to address this difficulty. In this respect, Spitsberg et al. [68] proved that casein and MFGM in buttermilk are associated via calcium bridges between phosphorylated casein and phosphate of PL. They used calcium-binding salts to dissociate these complexes into their components and to improve substantially the recovery of MFGM from buttermilk. Moreover, a considerable amount of PL has been considered to migrate to the cheese water soluble extract [69] despite the fact that the methods for their estimation are based on lipid extraction.

### 3.5. Particle Size Distribution

**Figure 4:**
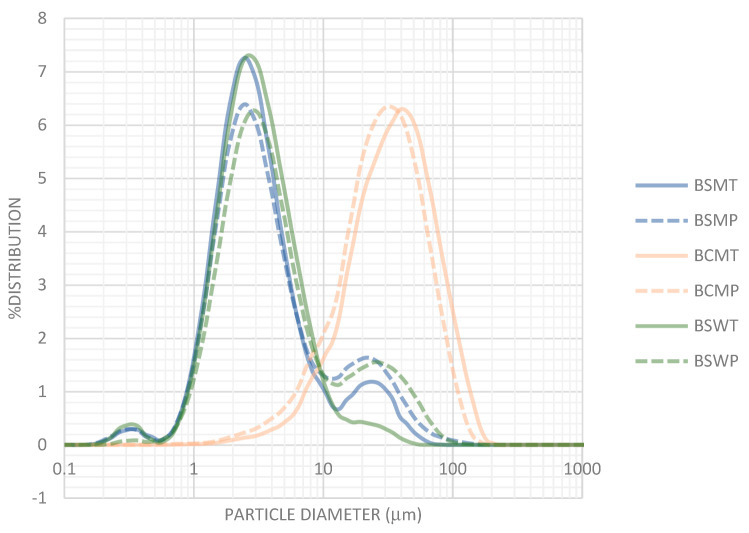
Particle size distribution in buttermilks. Mean percentages of three experiments. BSMT—buttermilk from SM-thermized cream; BCMT—buttermilk from CM-thermized cream; BSWT—buttermilk from SW-thermized cream; BSMP—buttermilk from SM-pasteurized cream; BCMP—buttermilk from CM-pasteurized cream; BSWP—buttermilk from SW-pasteurized cream. T—cream thermized at 68 °C for 10 min; P—cream pasteurized at 68 °C for 30 min.

The distribution of particle size in various types of buttermilk are shown in Figure 4. Particles with diameter >1 μm and maximum at 2.5–2.8 μm predominated in BSM and BSW, both of sheep origin. Nanoparticles with diameter 0.2–0.5 μm that can be assigned to casein micelles were also observed. Pasteurization increased the volume of particles with diameter >15 μm. On the other hand, particles in BCM with diameters greater than 10 μm, with maximum at 35–38 μm indicate the existence of aggregates or structures. Up to a point this difference can be assigned to the greater size of cow MFG compared with its sheep counterpart [34,35].

Relevant literature information is for buttermilk of cow origin. Morin et al. [26] report the existence of MFGM fragments with a size ranging from 0.1 to 2–3 μm and of structures consisted of caseins trapped in folded MFGM fragments. Particles with a diameter greater than 10 μm have been observed by Lambert et al. [51] in industrial whole buttermilk in very variable quantities, e.g., 6 ± 21% of total particles. These particles were large MFG resulted from the coalescence of small globules, aggregated MFG—i.e., butter fines—or flexible and folded fragments of MFGM.

### 3.6. Functional Properties

**Figure 5:**
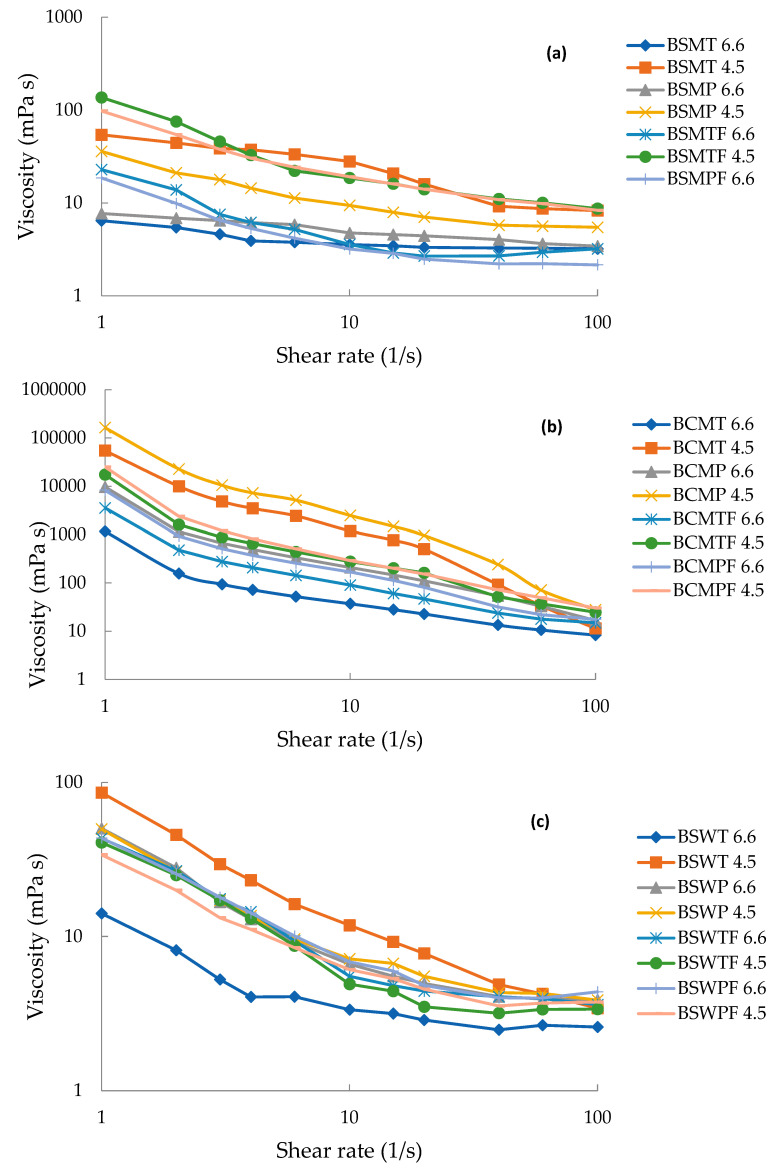
Viscosity (mPa·s) of buttermilk from sweet sheep cream (**a**), sweet cow cream (**b**) and sheep whey cream (**c**) at different pH. BSMT—buttermilk from SM-thermized cream; BCMT—buttermilk from CM-thermized cream; BSWT—buttermilk from SW-thermized cream; BSMP—buttermilk from SM-pasteurized cream; BCMP—buttermilk from CM-pasteurized cream; BSWP—buttermilk from SW-pasteurized cream. T—cream thermized at 68 °C for 10 min; P, cream pasteurized at 68 °C for 30 min. F at the end of a buttermilk code indicates the use of frozen cream.

All three experimental factors had a statistically significant effect on the viscosity (mPa·s) of buttermilks, presented in Table 6 and Figure 5. The substantially higher viscosity values of BCM are in accordance with the presence of large particles in the profile of Figure 4. Moreover, at pH 6.6 that is close to typical pH of sweet buttermilk, the more intense heat treatment significantly increased (*p* < 0.05) the viscosity of BCM and BSW opposite to BSM, which had the highest INSP content. Apparently, heat-induced complexation of serum proteins on the surface of the casein micelles of BSM did not significantly affect their size. The high concentration of casein of BSM indicates an abundance of potent complexation sites (Table 3, Figure 2). At pH 4.6, the viscosity of both sweet cream buttermilks was substantially increased due to the formation of casein aggregates. The increase was not significant (*p* > 0.05) for BSW due to low casein concentration.

The remaining physicochemical properties of buttermilks are shown in Table 7, grouped by the origin and storage of cream. According to multifactor ANOVA, the heat treatment of cream did not affect statistically significantly the physicochemical properties of buttermilks. The churning of frozen cream significantly decreased (*p* < 0.05) the FA at pH 6.6 and pH 4.5 and the PS at pH 6.6.

Regarding PS, as seen from Table 7, it varied from ~23 to 92%. For both ways of cream preservation, BCM with a pH of 6.6 exhibited high PS values, i.e., 90 and 92%, for non-frozen and frozen creams, respectively. BSM from non-frozen sweet cream also presented high PS value (92%). For BSM and BCM, both non-frozen and frozen, PS decreased significantly, e.g., from ~92 to ~23% for the BCMF, as the pH was lowered to 4.5, showing a great pH dependence. PS of BSW, for both pHs, was in the area of 59–67%, suggesting that the pH was not as important as for the rest of the samples.

The lower solubility at pH values lower than 5, observed for the creams of the present study, has also been reported in the literature [2], and it was attributed to the different contents of the samples in the insoluble at acidic pH casein and the mainly soluble whey protein. BSW contained a very low quantity of casein (Section 3.3, Table 3); therefore, the pH 4.6 did not affect its solubility.

The next functional property studied was ES (%). ES was in all cases greater for pH 4.5. This pH effect can be connected to the presence of INSP. Keeping in mind that whey has a very low INSP (Table 3), someone would expect that BSW will present the same ES for both pH values. However, that only occurred for the buttermilk from frozen cream. Regarding the origin of the sweet cream, buttermilks from cow non-frozen and frozen creams led to less stable emulsions when prepared at pH 6.6, having an ES of ~16 and 17%, respectively. For pH 4.5, the emulsion with the buttermilk from SW frozen cream was the less stable one (ES: ~30%). The emulsifying properties of buttermilks are attributed to their protein content as well as the presence of MFGM and their content of PL. Phan et al. [32] studied the emulsifying properties of MFGM materials and reported that the presence and concentration of all MFGM components (polar lipids, whey proteins, caseins, MFGM-specific proteins, and minerals), along with their possible interactions were critical for emulsion formation and stability. As reported earlier (Section 3.4.3), all buttermilks share statistically similar PL content. Moreover, BSW from both non-frozen and frozen creams had the lowest protein content, whereas BSM had the greatest (Table 2). However, for frozen creams, as mentioned earlier, proteolysis—due to additional treatments of cream before churning, and changes on casein micelles over freezing—may have occurred. A reduction in the micelles’ size affects the concentration of β-casein and thus, the surface tension. The total protein/PL ratio is often used to evaluate ES [70]. In the present study, this ratio was significantly affected only by the cream origin. It was statistically significantly higher (*p* < 0.05) in BSM; on average, it was 146 ± 32.4, 118 ± 64.5 and 94 ± 49.5 for BSM, BCM and BSW, respectively. No direct correlation of the various ratios of the samples of the present work to ES was found. On the same topic, the literature reports possible interactions between PL and proteins that stabilize or destabilize emulsions. For example, it was observed that interactions between proteins and PL at the air–water interfaces that they stabilize, are influenced by pH, which led to different interfacial structure [71]. The emulsification technique is also critical for MFGM emulsions, as reported by Jukkola et al. [72]. The particles with greater size (Figure 3) of BCM may also play a role for the low emulsion stability it exhibited.

FA was also determined by measuring the volume of the created foam for 30 mL of sample. Overall, values ranged from 0.04 to 0.4. In accordance to ES (%), FA was, for most of the samples, greater for pH 4.5. BSW from frozen cream (BSWF), presented a distinctive low FA value for both pH values, i.e., 0.042 and 0.055 for pH 6.6 and 4.6, respectively, while they exhibited the highest FA when coming from a non-frozen cream (i.e., 0.327 and 0.375 for pH 6.6 and 4.6, respectively). FA is related to a number of factors like the proteins’ solubility, size, flexibility and denaturation, the surface charges and its hydrophobicity, as well as the fat content of the buttermilks. Fat–protein interactions are known to decrease the FA of a protein solution, due to the fat’s amphiphilic nature and ability to displace the protein from the surface [73]. The PL content is another parameter affecting the creation of foam. As mentioned previously, BSW had the lowest protein to PL ratio.

PS is positively correlated with FA; however, in the present work, high PS values were not accompanied by a great FA. Thus, other factors should be taken under account. Protein denaturation results in greater surface hydrophobicity due to the unfolding of the proteins. According to Townsend and Nakai [74], the unfolding of denatured proteins in order to interact with the air–water interface, and its extent is critical for FA. Its increase leads to increased FA. Moreover, the ratio of casein, whey and MFGM protein affects protein absorption at the interface [75]. As already mentioned, in the present study, denaturation induced by the thermal processing was observed in BSW. The changes occurring during the freezing and storage of the frozen cream should also be taken under consideration.

As a step towards better understanding of our findings, multiple variable analysis was performed and exhibited interesting correlations between various protein-related indices and the studied functional properties (Table 8). WSN and its major constituents α-LA and β-LG were significantly correlated with all measured properties, with the exception of FA. To further investigate these correlations, a differential assessement was performed. Subsets of correlations are presented in Table 8. The NF subset did not include buttermilks from frozen creams to avoid the effect of changes occurred due to storage conditions. The SC subset did not include BSW to exclude any possible inteference of its totally different protein profile.

From Table 8, is evident that FA of buttermilks was not correlated statistically significantly with the nitrogenous fractions in contrast to viscosity and PS. The viscosity of the subset SC was correlated significantly with INSP, that is, mostly casein, since casein is present in the form of particles at native pH or as aggregates forming under acidic conditions. According to the linear correlation coefficients (Table 8), the INSP was very important for the PS of the various types of buttermilks at both pH values. This is an expected correlation due to the well-known behaviour of casein.

The only significant correlation of ES (%) with the INSP—in which casein predominates—was observed for subset SC at both pH values (Table 8). It can be assigned to the emulsification ability of caseins, in particular, that of β-casein [76]. Kim et al. [77] found that the concentration of β-LG correlated strongly and positively with the functional properties of whey protein concentrates; whereas, significant correlations with other whey proteins were not observed. Casein and whey proteins showed no synergistic effect to interface and emulsifying properties and competitive adsorption of β-casein and β-LG takes place [76,78]. In particular, β-casein directly reduces the surface tension at the interfaces, the serum proteins contribute to the formation of a “tight” viscoelastic structure at the interfaces enhancing stability, while the glycoproteins and PL of the membrane contribute with their amphiphilic structures [3]. SM contains more casein and β-casein contributes more to sheep casein fraction compared with its cow counterpart. The abundance of β-casein and β-LG in SM [34,35] can explain the significantly higher ES of BSM than that of BCM (Table 7). The correlation between ES and INSP was more intense at pH 4.5 than in native pH 6.6. Caseins in the micellar form, as happens at pH 6.6, are less surface active because the hydrophobic moieties are burned on the inside of the structure [70].

## 4. Conclusions

Under similar manufacturing conditions, BSM was more advantageous compared with its cow counterpart in regard to protein content and profile, biofunctional potential and functional properties. Moreover, the churning of frozen sweet sheep cream had marginal effects on the properties and behaviour of buttermilk, which is of great importance considering the limited production period of SM. The properties of buttermilk from SW were differentiated due to the predominance of lactose and soluble proteins. According to the results, buttermilk from the further exploitation of SW may have specific applications, e.g., as an ingredient in low-pH food formulae. The findings of the present study exhibited the potential of SM by-products and can be the basis for new research efforts on this topic.

## Figures and Tables

**Table 1:** Parameters of the manufacture of various types of buttermilk. Mean and standard deviation (sd) of six experiments. BSM—buttermilk from sheep milk cream; BCM—buttermilk from cow milk cream; BSW—buttermilk from sheep cheese whey cream; F at the end of a code indicates the use of frozen cream.

Cream	Churning (min)		Final T (°C)		Yield (%)	
	Mean	sd	Mean	sd	Mean	sd
Non-frozen						
BSM	14.75 b	0.61	7.67 b	0.31	47.84	3.12
BCM	17.83 c	1.13	10.42 c	0.85	48.54	0.48
BSW	9.71 a	0.83	6.8 a	0.45	48.46	0.72
Frozen						
BSMF	1.0 a *	0.16	6.45 a *	0.25	41.58 a *	1.78
BCMF	4.54 b *	2	6.48 a *	0.29	44.79 b *	0.65
BSWF	1.67 a *	0.58	6.47 a	0.05	44.45 b *	2.86

a–c—statistically significant differences (LSD, *p* < 0.05) within the group of non-frozen or frozen cream; *—significant difference (LSD, *p* < 0.05) between buttermilks of the same type from non-frozen or frozen cream.

**Table 2:** Composition of various types of buttermilk. Mean and standard deviation (sd) of six experiments. BSM—buttermilk from sheep milk cream; BCM—buttermilk from cow milk cream; BSW—buttermilk from sheep cheese whey cream; F at the end of a code indicates the use of frozen cream; LA—lactic acid; TS—total solids; SNF—solids (non-fat).

Cream							FTIR Analysis											
	pH		%LA		%Fat ^1^		%Fat		%Protein		%Lactose		%TS		%SNF		P/F	
	Mean	sd	Mean	sd	Mean	sd	Mean	sd	Mean	sd	Mean	sd	Mean	sd	Mean	sd	Mean	sd
Non-frozen																		
BSM	6.75 b	0.03	0.20 c	0.01	2.78 c	0.2	2.86 c	0.23	5.51 c	0.09	5.04 a	0.05	13.98 c	0.30	11.11 c	0.10	1.93 b	0.16
BCM	6.72 b	0.17	0.13 b	0.01	0.91 a	0.35	1.02 a	0.29	3.28 b	0.07	4.99 a	0.08	10.34 b	0.26	9.35 b	0.12	3.42 c	0.91
BSW	6.43 a	0.04	0.11 a	0.004	1.99 b	0.32	2.21 b	0.35	2.48 a	0.02	5.33 b	0.12	9.79 a	0.35	8.27 a	0.12	1.15 a	0.2
Frozen																		
BSMF	6.55 a,b *	0.04	0.18 c	0.01	1.81 b *	0.31	1.95 b *	0.28	5.49 c	0.23	5.20 b *	0.09	13.22 c *	0.48	11.25 c	0.21	2.86 b *	0.37
BCMF	6.61 b	0.07	0.13 b	0.01	3.36 c *	0.63	3.39 c *	0.66	3.06 b *	0.08	4.81 a *	0.10	12.29 b *	0.57	8.94 b *	0.14	0.93 a *	0.19
BSWF	6.49 a	0.07	0.11 a	0.01	0.84 a *	0.25	1.03 a *	0.29	2.43 a	0.07	5.27 b	0.12	8.49 a *	0.32	8.15 a	0.14	2.62 b *	1.15

^1^ estimated by the Gerber method; a–c—statistically significant differences (LSD, *p* < 0.05) within the group of non-frozen or frozen cream; *—significant difference (LSD, *p* < 0.05) between buttermilks of the same type from non-frozen or frozen cream.

**Table 3:** Nitrogenous fractions of various types of buttermilk. Mean and standard error (*se*) of the experiments (*n*). BSM—buttermilk from sheep milk cream; BCM—buttermilk from cow milk cream; BSW—buttermilk from sheep cheese whey cream; NF and F—buttermilk from non-frozen and frozen cream, respectively; T—cream thermized at 68 °C for 10 min; P—cream pasteurized at 68 °C for 30 min; INSP—protein insoluble at pH 4.6; SP—soluble proteins; WSN—(water)-soluble nitrogen at pH 4.6; TN—total nitrogen; β-LG—native β-lactoglobulin; α-LA—native α-lactalbumin; CMP—caseinomacropeptide.

Cream	Kjeldahl Analysis			RP-HPLC Profile			
	%INSP	%SP ^2^	WSN/TN	β-LG, mg/L	α-LA, mg/L	CMP, mg/L	β-LG/α-LA
Origin (*n* = 12)							
BSM	3.74 c	1.37 b	26.75 a	6407 b	2220 b		2.92 b
BCM	2.08 b	1.0 a	32.46 b	3057 a	1401 a		2.19 a
BSW	0.29 a	1.65 c	85.36 c	6747 c	2179 b	2348	3.12 b
*se*	0.04	0.03	1.27	84.7	49.7		0.09
Heat treatment (*n* = 18)							
T	1.96 a	1.39 b	51.03 b	5582 b	1984	2378 ^1^	2.75
P	2.12 b	1.29 a	45.35 a	5227 a	1882	2318 ^1^	2.73
*se*	0.03	0.03	1.04	69.2	40.46	65.7	0.07
Storage (*n* = 18)							
NF	2.14	1.29 a	45.07 a	5273	1883	2575 ^1^ b	2.74
F	1.94	1.39 b	51.31 b	5536	1984	2122 ^1^ a	2.69
*se*	0.03	0.03	1.035	69.2	40.46	65.7	0.07

^1^ *n* = 6; ^2^ WSN × 6.38; a–c—statistically significant differences (LSD, *p* < 0.05) within the means of each experimental factor.

**Table 4:** Major minerals (mg/100 g buttermilk) of various types of buttermilk. Mean and standard deviation (sd) of 6 experiments. BSM—buttermilk from sheep milk cream; BCM—buttermilk from cow milk cream; BSW—buttermilk from sheep cheese whey cream; F at the end of a buttermilk code indicates the use of frozen cream.

Cream	Ca		Mg		K		Na		P	
	Mean	sd	Mean	sd	Mean	sd	Mean	sd	Mean	sd
Non-frozen										
BSM	113.28 c	4.66	20.55 b	0.71	112.45 a	5.19	76.63 a	4.52	120.28 c	4.07
BCM	57.03 b	3.22	13.73 a	0.87	149.25 c	14.40	75.23 a	7.45	79.77 b	3.76
BSW	22.42 a	1.37	13.48 a	0.82	130.43 b	9.94	91.77 b	5.05	46.7 a	3.18
Frozen										
BSMF	109.48 c	4.33	20.93 b	1.14	125.73 a	5.35	77.25 a	7.24	117.77 c	7.43
BCMF	52.72 b	5.45	12.92 a	0.71	146.85 b	7.96	81.35 a,b	5.32	76.07 b	2.72
BSWF	23.7 a	2.35	13.95 a	0.76	119.3 a	9.34	86.85 b	7.94	40.6 a *	2.92

a–c—statistically significant differences (LSD, *p* < 0.05) within the groups of non-frozen or frozen cream; *—significant difference (LSD, *p* < 0.05) between buttermilks of the same type from non-frozen or frozen cream.

**Table 5:** Antioxidant potential of various types of buttermilk. Mean and standard deviation (sd) of six experiments. BSM—buttermilk from sheep milk cream; BCM—buttermilk from cow milk cream; BSW—buttermilk from sheep cheese whey cream; F at the end of a buttermilk code indicates the use of frozen cream.

Cream	%DPPH∙ Radical Scavenging Activity (RSA)		%Fe^2+^ Chelating Activity (CA)	
	Mean	sd	Mean	sd
Non-frozen				
BSM	79.04 b	2.79	84.14 c	2.92
BCM	62.95 a	2.64	74.69 b	6.42
BSW	81.10 b	4.30	17.80 a	4.43
Frozen				
BSMF	73.90 c	5.20	90.53 c *	1.01
BCMF	60.56 a	3.50	66.54 b *	1.76
BSWF	67.115 b *	3.13	17.36 a	8.68

a–c—statistically significant differences (LSD, *p* < 0.05) within the groups of non-frozen or frozen cream; *—significant difference (LSD, *p* < 0.05) between buttermilks of the same type from non-frozen or frozen cream.

**Table 6:** Viscosity (mPa·s) of various types of buttermilk at different pH. Mean and standard deviation (sd) of three experiments. BSM—buttermilk from sheep milk cream; BCM—buttermilk from cow milk cream; BSW—buttermilk from sheep cheese whey cream; T—cream thermized at 68 °C for 10 min; P—cream pasteurized at 68 °C for 30 min; F at the end of a buttermilk code indicates the use of frozen cream.

Cream	Viscosity (mPa·s) pH 6.6				Viscosity (mPa·s) pH 4.5			
	Thermized (T)		Pasteurized (P)		Thermized (T)		Pasteurized (P)	
	Mean	sd	Mean	sd	Mean	sd	Mean	sd
Non-frozen								
BSM	3.21 a	0.132	3.43 a	0.24	8.28 b	0.46	5.48 a #	0.38
BCM	8.33 b	0.487	16.79 b #	1.22	11.46 c	0.71	27.91 b #	1.67
BSW	2.58 a	0.044	3.89 b #	0.16	3.42 a	0.31	3.86 a	0.37
Frozen								
BSMF	3.21 a	0.3	2.16 a	0.15	8.68 b *	0.28	8.34 b	0.17
BCMF	14.99 b *	1.27	17.68 b #	0.90	24.80 c *	2.35	30.41 c #	2.36
BSWF	3.61 a *	0.142	4.37 c # *	0.48	3.38 a	0.28	3.75 a	0.36

a–c—statistically significant differences (LSD, *p* < 0.05) within the groups of non-frozen or frozen cream; *—significant difference (LSD, *p* < 0.05) between buttermilks of the same type from non-frozen or frozen cream; #—significant difference (LSD, *p* < 0.05) between buttermilks of the same type from thermized or pasteurized cream.

**Table 7:** Physicochemical properties of various types of buttermilk. Mean and standard deviation (sd) of six experiments. BSM—buttermilk from sheep milk cream; BCM—buttermilk from cow milk cream; BSW—buttermilk from sheep cheese whey cream; F at the end of a buttermilk code indicates the use of frozen cream.

	%Emulsion Stability (ES)				Foaming Ability (FA)				%Protein Solubility (PS)			
Cream	pH 6.6		pH 4.5		pH 6.6		pH 4.5		pH 6.6		pH 4.5	
	Mean	sd	Mean	sd	Mean	sd	Mean	sd	Mean	sd	Mean	sd
Non-frozen												
BSM	20.67 b	2.90	56.09	7.87	0.235 a	0.044	0.242	0.018	92.08 b	7.48	26.10 a	3.11
BCM	15.86 a	0.81	50.64	9.06	0.235 a	0.049	0.315	0.223	89.8 b	4.64	25.42 a	1.29
BSW	19.55 b	1.45	49.44	2.32	0.327 b	0.079	0.375	0.074	66.8 a	2.26	67.04 b	6.37
Frozen												
BSMF	19.21	1.85	54.76 b	2.74	0.182 b	0.070	0.352 c *	0.098	79.94 b *	8.46	34.01 b	8.93
BCMF	17.0 *	1.37	54.54 b	1.02	0.252 c	0.052	0.24 b	0.060	91.6 c	1.65	23.4 a *	1.50
BSWF	20.16	3.97	29.84 a *	2.69	0.042 a *	0.017	0.055 a *	0.006	62.88 a	3.42	58.83 c *	1.13

a–c—statistically significant differences (LSD, *p* < 0.05) within the groups of non-frozen or frozen cream; *—significant difference (LSD, *p* < 0.05) between buttermilks of the same type from non-frozen or frozen cream.

**Table 8:** Linear correlation coefficients between nitrogenous fractions and functional properties of buttermilks. V—viscosity (mPa⋅s); ES—emulsion stability (%); FA—foaming ability; PS—protein solubility (%); INSP—protein insoluble at pH 4.6; WSN—(water)-soluble nitrogen at pH 4.6; β-LG—native β-lactoglobulin; α-LA—native α-lactalbumin; NF—group of buttermilks from non-frozen cream; SC—group of buttermilks from sweet cream.

Properties		INSP, %	WSN, %	α-LA, mg/L	β-LG, mg/L	β-LG/α-LA
V-pH 6.6	total ^1^	−0.03	−0.73	−0.89	−0.91	−0.71
	NF ^2^	0.08	−0.78	−0.86	−0.90	−0.71
	SC ^3^	−0.89	−0.74	−0.90	−0.92	−0.76
V-pH 4.5	total	0.18	−0.74	−0.83	−0.89	−0.73
	NF	0.22	−0.77	−0.82	−0.81	−0.64
	SC	−0.83	−0.64	−0.81	−0.86	−0.74
ES-pH 6.6	total	0.03	0.50	0.41	0.58	0.64
	NF	0.20	0.72	0.70	0.88	0.84
	SC	0.66	0.67	0.62	0.78	0.81
ES-pH 4.5	total	0.18	−0.74	−0.83	−0.89	−0.73
	NF	0.22	−0.77	−0.82	−0.81	−0.64
	SC	−0.83	−0.64	−0.81	−0.86	−0.74
FA-pH 6.6	total	0.12	−0.21	−0.27	−0.36	−0.32
	NF	−0.60	0.58	0.33	0.28	0.20
	SC	−0.32	−0.32	−0.41	−0.35	−0.19
FA-pH 4.5	total	0.24	−0.14	0.01	−0.18	−0.28
	NF	−0.44	0.24	0.17	0.10	0.03
	SC	0.05	0.03	−0.41	0.05	−0.10
PS-pH 6.6	total	0.71	−0.71	−0.52	−0.59	−0.51
	NF	0.85	−0.64	−0.45	−0.25	−0.03
	SC	−0.26	−0.21	−0.18	−0.14	−0.09
PS-pH 4.5	total	−0.76	0.83	0.62	0.68	0.62
	NF	−0.89	0.84	0.51	0.47	0.36
	SC	0.55	0.44	0.61	0.50	0.31

^1^—all experimental buttermilks from non-frozen and frozen creams; ^2^—buttermilks from non-frozen creams; ^3^—buttermilks from sweet sheep and cow cream.

## Data Availability

The data is presented in the manuscript.

## Abbreviations

α-LA	native α-lactalbumin
β-LG	β-lactoglobulin
AAS	atomic absorption spectrometric method
ANOVA	analysis of variance
BCM	buttermilk from cow milk sweet cream
BCMPF	buttermilk from CM-pasteurized and frozen cream
BCMTF	buttermilk from CM-thermized and frozen cream
BSM	buttermilk from sheep milk sweet cream
BSMPF	buttermilk from sheep—pasteurized and frozen cream
BSMTF	buttermilk from sheep—thermized and frozen cream
BSW	buttermilk from sheep whey cream
BSWPF	buttermilk from sheep whey—pasteurized and frozen cream
BSWTF	buttermilk from sheep whey—thermized and frozen cream
CA	chelating activity
CM	cow milk
CMP	caseinomacropeptide
DPPH	2,2-diphenyl-1-picrylhydrazyl
EDTA	ethylenediaminetetraacetic acid
ES	% emulsion stability
F	frozen cream
FA	foaming ability
INSP	protein insoluble at pH 4.6
LA	lactic acid
LSD	least significance difference
MAS	molecular absorption spectrometry
MFG	milk fat globule
MFGM	milk fat globule membrane
N	nitrogen
NF	non-frozen cream
P	pasteurized cream
PL	phospholipids
PVDF	poly(vinylidene fluoride) (filter)
PS	% protein solubility
RP-HPLC	reversed-phase high-performance liquid chromatography
RSA	radical scavenging activity
sd	standard deviation
se	standard error
SM	sheep milk
SNF	non-fat solids
SW	sheep cheese whey
T	thermized cream
TN	total nitrogen
WSN	water-soluble nitrogen
